# A Matron on "Other People's Money"

**Published:** 1888-10-20

**Authors:** 


### A MATRON ON "OTHER PEOPLE'S MONEY."

A provincial matron writes: Your remarks on "Other People's Money" are only too true, but all the reckless waste is not due to matrons and secretaries alone. Waste will go on to a great extent until each hospital worker realises the full meaning of trustfulness and faithfulness in small matters as well as great.

No matron can altogether prevent the waste of bread, meat, potatoes, etc. Patients are so afraid of not getting "their due," that they would rather leave half their food than say, "Nurse, I can't eat it. Can I have it for supper?"

The H.S. also is often thoughtlessly extravagant with dressings, lint, tow, wool, and drugs, to say nothing of food and gas. The latter often burns for hours when the doctor is out, and it might just as well be lowered. As a pro., I have often seen large pieces of lint used as dishcloths, polishers, and for washing patients, the excuse being, "Oh! they have brought nothing in with them."

There are many of us, I trust, who do truly try to make money and stores go as if they were our own, without any expectation of even commendation (I suppose a little praise would spoil us, for I may say we never receive it). But until each one will "sweep his (or her) own door-step," because it is a Christian duty so to do, waste will go on in wards and kitchens, gas and other bills will be too high, and the matron and secretary will be "sat upon" on finance days for the faults of others.

